# Effect of Co-presentation of Adhesive Ligands and Short Hyaluronan on Lymphendothelial Cells

**DOI:** 10.3389/fbioe.2018.00025

**Published:** 2018-03-23

**Authors:** Christiane H. Antoni, Yvonne McDuffie, Jochen Bauer, Jonathan P. Sleeman, Heike Boehm

**Affiliations:** ^1^Department of Cellular Biophysics, Max Planck Institute for Medical Research, Heidelberg, Germany; ^2^Department of Biophysical Chemistry, University of Heidelberg, Heidelberg, Germany; ^3^Institute of Toxicology and Genetics, Karlsruhe Institute for Technology (KIT), Karlsruhe, Germany; ^4^Medical Faculty Mannheim, University of Heidelberg, Mannheim, Germany

**Keywords:** hyaluronan, lymphangiogenesis, extracellular matrix mimetic, lymphendothelial cells, bioactive interface

## Abstract

Controlled activation of lymphangiogenesis through functional biomaterials represents a promising approach to support wound healing after surgical procedures, yet remains a challenge. In a synthetic biological approach, we therefore set out to mimic the basal microenvironment of human primary dermal lymphatic endothelial cells (LECs) during lymphangiogenesis. As the extracellular matrix component hyaluronan (HA) regulates lymphangiogenesis, we designed a bifunctional surface in which adhesive peptide ligands and short HA oligosaccharides (sHA) tethered to nanoparticles are copresented to the basal side of LECs in a controlled, concentration-dependent manner. Exposure of LECs to sHA in solution to mimic luminal stimulation of the cells did not result in modified metabolic activity. However, LECs grown on the bifunctional adhesive surfaces showed a biphasic change in metabolic activity, with increased metabolic activity being observed in response to increasing nanoparticle densities up to a maximum of 540 particles/μm^2^. Thus, interfaces that concomitantly present adhesive ligands and sHA can stimulate LEC metabolism and might be able to trigger lymphangiogenesis.

## Introduction

Lymphangiogenesis, the sprouting of new lymph vessels, is a vital process during embryogenesis, tumor growth, and wound healing (Martínez-Corral et al., 2012). It correlates with significant changes in the composition of the extracellular matrix (ECM) consisting mainly of proteins, glycosaminoglycans, and water. The RGD motif an evolutionarily conserved three amino-acid sequence built up of arginine, glycine, and aspartate is present in various ECM proteins, e.g., fibronectin, vitronectin, and fibrinogen (Mecham, 2011). This sequence is recognized by a subset of the integrins in the cell membrane. Over the last years, several techniques have been developed to modify biomaterial surfaces with the RGD motif as a so-called adhesive ligand to investigate receptor-mediated cell interactions (Rahmany and van Dyke, 2013). Furthermore, lymphangiogenesis is connected to the ECM compound hyaluronan (HA). Especially different natural occurring fragments show different effects on cells (Schmaus et al., 2014; Yu et al., 2015). HA is composed of repeating disaccharide units of *N*-acetyl-d-glucosamine and d-glucuronic acid, and is synthesized with a molecular weight of 10^3^–10^4^ kDa, which corresponds to 2,000–25,000 disaccharide units and a total contour length of 2–25 µm (Winter et al., 1975; Scott et al., 1991; Jackson, 2009). Through enzymatic degradation and oxidative stress reactions, HA can be cleaved into various sizes, which differ in their biological activity (as reviewed in Bohaumilitzky et al., 2017). High molecular weight HA inhibits proliferation, migration, and has anti-angiogenic and anti-inflammatory effects on blood endothelial cells (Mo et al., 2011; Atta et al., 2012; Ghazi et al., 2012; Anderegg et al., 2014; Tolg et al., 2014). On the other hand, low molecular weight HA can stimulate proliferation, motility, and tube formation in endothelial cells and promotes inflammation and angiogenesis (Stern et al., 2006; Mo et al., 2011; Du et al., 2013; Tolg et al., 2014). Interestingly, the cellular response to short, low molecular weight HA (sHA) is also concentration dependent. Thus, the application of sHA with 4–20 repeating units on blood vessel cells stimulates proliferation in a concentration range of 3–20 µg/mL showing a maximum response at 10 µg/mL (Mo et al., 2011). This biphasic effect on the proliferation of primary human dermal lymphatic endothelial cells (LECs) was also found for sHA with 4–13 disaccharide units in solution, with maximal effect on proliferation observed at a concentration of 5 µg/mL (Bauer et al., 2018).

**Figure 1:**
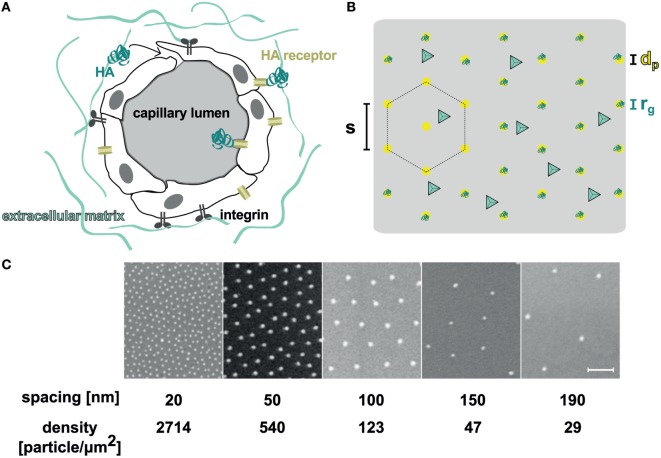
**(A)** Scheme of the possible sites where interactions between hyaluronan (HA) and lymphatic capillaries take place. The HA receptor such as LYVE-1, CD44, or RHAMM is located on both the luminal and basal surfaces of the cells. **(B)** The orthogonal functionalized surface used in these experiments was passivated with polyethylene glycol, which was further modified with cyclic RGD ligand (light blue tetrahedron). The nanostructures had a particle diameter of *d*_p_ = 10 nm, and were functionalized with HA oligosaccharides [turquoise, radius of gyration *r*_g_ = 7.1 nm (Kuehl et al., 2016)]. The hexagonal packed particles had variable interparticle spacings (s). **(C)** SEM pictures of the nanostructured surfaces with different interparticle distances. Scale bar corresponds to 100 nm.

To date, sHA stimulation experiments usually rely on the administration of sHA in solution to adherent cells. This experimental setup corresponds to the stimulation of sHA on the luminal side of the lymph vessel. In contrast, endothelial cells *in vivo* are also in direct contact to HA on their basal side. So far, it has been difficult to study the effect of sHA specifically administered to the basal side of LECs in a defined manner *in vitro*, due to the lack of appropriate copresentation techniques for HA and adhesive ligands. To create a bifunctional surface different immobilization strategies can be used. On the one hand, it is possible to immobilize the two molecules of interest statistically distributed on the surface. On the other hand, more defined methods are described using nanostructured surfaces which enable the creation of well-controlled patterns on the surface followed by the binding of the molecule of interest *via* strong chemical interactions. This can be combined with either nanoparticles made from another metal and so another binding partner or a chemical immobilization strategy such as copper-catalyzed click reaction (Schenk et al., 2014; Guasch et al., 2016). In a synthetic biological approach, we therefore set out to mimic the basal microenvironment of LECs during lymphangiogenesis. To this end we designed an ECM model in which adhesive ligands and sHA are copresented to the basal side of LECs in a controlled, concentration-dependent manner. Using this setup, we aimed in this study to investigate differences in the metabolic response of LECs after exposure to sHA on either their basal and luminal sides (Figure 1A).

## Materials and Methods

### Derivation of sHA

Short HA with a weight range from 10 to 20 kDa (25- to 50-mer) produced by a heat fragmentation of high molecular weight HA was obtained from Lifecore Biomedical. Enzymatically digested sHA with an average weight <10 kDa (25-mer) was produced from Healon 5 (Abbott Medical Optics), a highly purified high molecular weight HA with an average weight of 4 MDa, as previously described (Bauer et al., 2018). Briefly, Healon 5 was dissolved at 5 mg/mL in 0.3 M sodium phosphate, pH 5.3, sonified, and subsequently enzymatically digested with 200 U/mL bovine testes hyaluronidase (Sigma-Aldrich) for 6 h at 37°C. The resulting fragments were centrifuged through an Amicon ultracentrifugal filter (Millipore) with a 10-kDa molecular weight cutoff. The flow through containing HA fragments <25 disaccharide units in length (Schmaus et al., 2014) was used for further experiments.

### Functionalization of sHA Species

Functionalization of HA with a thiol group was performed as previously described (Minsky et al., 2016). Briefly, the HA was stirred with sodium cyanoborohydride (Sigma-Aldrich) and cysteamine hydrochloride (Sigma-Aldrich) for 5 days, then purified by dialysis. The product was recovered by freeze drying. The characterization of the chemical modified sHA is found in the Supplementary Material (Minsky et al., 2016).

### Surface Preparation Using Block-Copolymer-Micellar Nanolithography (BCMN)

Nanostructured surfaces were prepared according to general block copolymer micellar nanolithography protocols (Boehm, 2008). Briefly, poly[styrene(*x*)-block-(2-vinylpyridine)(*y*)] [PS(*x*)-*b*-P2VP(*y*)] (Polymer Source, Canada) was dissolved in ortho-xylene (Merck) to a final concentration of 2–8 mg/mL (Table 1) in a precleaned glass tube (ThermoFischer). After 24 h stirring, tetrachloroaureate(III)trihydrate (HAuCl_4_ × 3 H_2_O, Sigma-Aldrich) was added and the solution was stirred for another 24 h.

**Table 1:** The change in the composition of styrene units (*x*) and vinylpyridin units (*y*) enables the preparation of nanostructured surfaces with different particle densities.

Particle density (particles/μm^2^)	*X*	*Y*	Concentration (mg/mL)	Speed (rpm)	Hexagonality (%)
29	5,348	713	2.0	3,000	48
47	5,348	713	2.0	4,000	50
123	1,056	671	3.0	3,000	63
540	1,056	671	8.0	4,000	68
2,714	154	33	5.0	4,000	60

*The table summarizes the applied polymer structures with the corresponding particle density, concentration of the used gold solution, and the speed of the spin coater. Also, the calculated hexagonality of the particles is shown here*.

The glass surfaces (*d* = 22 mm, Menzelglaeser) were cleaned over night with a 3:1 mixture of freshly prepared Caro’s acid [H_2_SO_4_ (Sigma-Aldrich) and H_2_O_2_ (30%, AppliChem)], then rinsed with ddH_2_O and dried under a nitrogen stream. The slides were fixed in a spin-coater (WS-400A-6NPP/LITE/8K, Laurell) under a slight vacuum and 20 µL of the micellular solution was quickly added. Each surface was spin coated for 30 s with a speed of 3,000–4,000 rpm (Table 1). The gold salt was reduced and the polymer was removed by treatment with hydrogen plasma (W10, 350 W, 0.4 mbar, 45 min; Plasma System 100-E, PVA TePla). The hexagonality of the nanoparticles and the distance between them was analyzed using SEM (Ultra 55 SEM, Zeiss) as described previously (Boehm, 2008). Surfaces used in this study had nanoparticle densities of about 2,714, 540, 123, 47, and 29 particles/μm^2^ with a hexagonality of at least 48%.

### Functionalization of Surfaces With Click-Polyethylene Glycol (PEG)

Nanostructured surfaces or Caro precleaned blank glass slides (*d* = 22 mm) were activated using oxygen plasma (150 W, 0.4 mbar, 10 min; Plasma System 360M, PVA TePla). To functionalize the surface with click-PEG, a 1:100 mixture of alkyne-PEG_3000_-silane [(EtO)_3_Si-(CH_2_)_3_-NH-C(O)-NH-PEG_3000_-NH-C(O)-(CH_2_)_2_-C≡CH] and PEG_2000_-silane [(EtO)_3_Si-(CH_2_)_3_-NH-C(O)-NH-PEG_2000_] was used. Therefore, a Schlenk flask is flushed with nitrogen and 30 mL dry toluene (Merck), 3 µL ddH_2_0, 3 drop of triethyl amine (Sigma-Aldrich), and a spatula tip of the PEG mixture were added. The synthesis of both PEG species is described elsewhere (Lohmuller et al., 2011; Schenk et al., 2014). The surfaces were inserted in the flask in a custom-made glass slide holder. The reaction solution was heated to 80°C over night. The glass slides were rinsed twice with ethyl acetate (Carl Roth) and sonicated for 1 min, then rinsed with methanol (Carl Roth) and ddH_2_O, and dried under a stream of nitrogen.

In a second step, cyclic RGD with an azide function [c(RGDfE)-KN_3_, PSL Peptide Specialty Laboratories GmbH] was clicked to the alkyne groups of the PEG using copper-catalyzed azide–alkyne cycloaddition. Surfaces were incubated with 75 µL TRIS-buffer (100 mM, pH 8.5, Sigma-Aldrich) containing 100 mM ascorbic acid (Sigma-Aldrich), 150 µM cRGD-azide, and 1 mM copper sulfate (Sigma-Aldrich) upside down on parafilm in a humidifying chamber for 2.5 h at room temperature. The surfaces were washed three times with ddH_2_O for 10 min each and dried under a stream of nitrogen.

### Cell Experiments

Human primary dermal LECs (order no. C12216, Batch 3061003.3, PromoCell) were applied were seeded in 5 mL EGM-2 MV medium (endothelial basal medium-2, Lonza) in a 25 cm^2^ cell culture flask (Greiner BioOne). The media contain 0.1% epidermal growth factor (hEGF), 0.1% vascular endothelial growth factor (VEGF), 0.1% R3-insulin-like growth factor (R3-IGF-1), 0.1% ascorbic acid, 0.04% hydrocortisone, 0.4% human fibroblast growth factor-beta (hFGF-β), 5.0% fetal bovine serum (FBS), and 0.1% Gentamicin/Amphotericin-B (GA, all EGM-2 MV Single Quots, Lonza). When the cells reached 80% confluency, the monolayer was washed twice with warm PBS (Gibco) before being incubated with trypsin-EDTA (Gibco) for 3 min. The cells were centrifuged for 5 min at 0.2× *g* and reseeded into new flasks as required.

### Soluble HA Species

The functionalized glass slides were washed steril with steril PBS and added under sterile conditions to a 12-well plate (Greiner BioOne). Stock solutions of the HA-species (40 µg/mL in medium) were prepared and diluted in the ratios of 1:20, 1:8, 1:4, and 1:2 to a final volume of 500 µL per well. In total, 50,000 cells in 500 µL medium were added to each well. This results in final HA concentrations of 1.0, 2.5, 5.0, and 20.0 µg/mL. The cells were incubated at 37°C and 5% CO_2_.

### Immobilized HA Species

To functionalize the gold nanoparticles on the surfaces after passivation, the surfaces were incubated upside down with 75 µL of an end-thiolated HA species [1wt%/v% in PBS (pH = 7.4)] on parafilm in a humidified chamber for 1 h at room temperature. The surfaces were rinsed twice with PBS and added to a 12-well plate under sterile conditions. In total, 50,000 cells were seeded per well in 1 mL medium and incubated at 37°C and 5% CO_2_.

### Determination of the Relative Metabolic Activity

After seeding for 46 h, cell culture medium was removed and 55 µL of the AlamarBlue kit (ThermoFischer) was added. After incubation for 2 h at 37°C and 5% CO_2_, fluorescence was measured with an excitation wavelength of 540 nm and an emission wavelength of 585 nm with a plate reader (Infinity M 200, Tecan). The results were normalized to the mean value of the background (AlamarBlue_c_, surfaces without applied HA). Experiments were carried out in triplicate.

After AlamarBlue measurements, the surfaces were washed twice with PBS and frozen at −80°C. CyQuant dye (400 × solution) and the cell lysis buffer (20 × solution) of the CyQuant kit (Thermofischer) were diluted with ddH_2_O, then 600 µL of the final solution were added to each surface and incubated for 5 min. The fluorescence was then measured with an excitation wavelength of 480 nm and an emission wavelength of 520 nm with a plate reader (Infinity M 200, Tecan). The results were normalized to the mean value of the background (CyQuant_c_, surfaces without applied HA).

The relative metabolic activity was calculated by dividing the normalized result for the metabolic activity for each surface by the corresponding normalized result of the CyQuant assay using Excel for Mac 2011 (version 14.7.1, Microsoft):

$$\text{rel}-\text{metabol.activity}=\frac{{\text{AlamarBlue}}_{x}/{\;}_{\bar{{\text{AlamarBlue}}_{c}}}}{{\text{CyQuant}}_{x}/{\;}_{\bar{{\text{CyQuant}}_{c}}}}\cdot 100.$$

The data were plotted and a Kruskal–Wallis test followed by Dunn’s multiple comparison test was performed using GraphPad Prism6 (for Mac, version 6.0e, GraphPad Software Inc., USA).

## Results

Lymphatic endothelial cells cannot grow directly on surfaces functionalized with only sHA, as they also require adhesive interactions to bind to surfaces. Using gold-nanostructured glass surfaces with tuneable inter-particle distances produced by block–copolymer–micellar nanolithography (BCMN; Lohmuller et al., 2011), we therefore developed an interface in which adhesive ligands and sHA are concomitantly presented in a precisely controlled manner (Figures 1B,C). The area between the nanoparticles was covalently functionalized with an inert PEG-based coating (Blummel et al., 2007). We further developed this coating to include a specific adhesive peptide (Schenk et al., 2014). Specifically, the glass surface was passivated with a mixture of PEG_2000_-silane and alkyne-PEG_3000_-silane (click-PEG) to prevent unspecific interactions between cells or proteins with the surfaces. LEC adhesion was subsequently facilitated by the attachment of azide-functionalized adhesive peptides to the alkyne groups of the click-PEG *via* copper-catalyzed azide–alkyne cycloaddition (CuAAC). Primary human LECs adhered best on surfaces functionalized with c(RGDfE) ligands (Kapp et al., 2017) and reasonably well on peptides with the amino-acid sequences GYIGSRY, SVVYGLR, GRGDSP, and REDV (Figure S2 in Supplementary Material). Adhesion comparable to cell culture plastic was achieved at a ratio of PEG_2000_-silane to alkyne-PEG_3000_-silane of 1:100 (Figure S3 in Supplementary Material).

In the next step, sHA was bound to the gold nanoparticles using sHA thiolated at its reducing end, which readily self-assembles on gold surfaces to form stable and bioactive adlayers (Minsky et al., 2016). On our surfaces, sHA only bound to the gold nanoparticles. Thus, the density of sHA in our experimental setup could be modulated by varying the distance between the nanoparticles, and thus their density on the surfaces (Figure 1B). Due to the size of the nanoparticles (*d*_p_ = 10 nm) compared with the radius of gyration of the HA species (*r*_g_ = 7.1 nm; Kuehl et al., 2016), we assumed that only one HA receptor is able to bind to one gold nanoparticle (Banerji et al., 2016). Before determining and comparing the influence of sHA on LECs using the different surfaces, it was verified that the cells were able to adhere to the surfaces in a comparable manner (see Supplementary Material).

**Figure 2:**
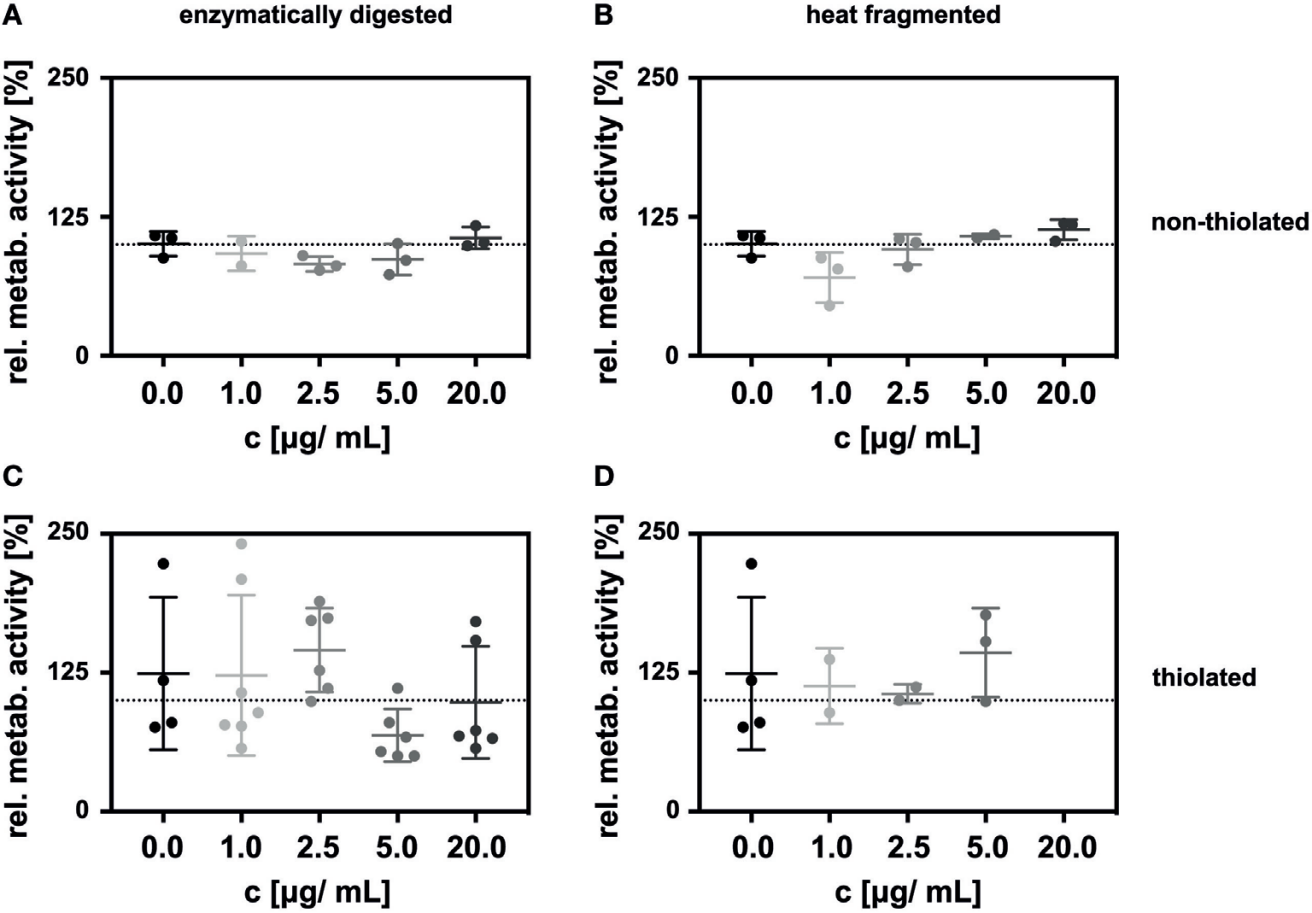
Incubation of lymphatic endothelial cells (LECs) with HA oligosaccharides (sHA) in solution had no impact on the relative metabolic activity of the cells. The graphs show the metabolic activity of LECs normalized to the amount of DNA on c(RGDfE) functionalized click-PEG surfaces after exposure to **(A)** enzymatically digested sHA and **(B)** heat-fragmented sHA and their respective thiolated species **(C,D)**. The Kruskal–Wallis test followed by the Dunn’s multiple comparison indicated no significant difference between the control and the different concentrations of the hyaluronan species.

In initial experiments, we employed a click-PEG coating, but on glass surfaces without any gold nanoparticles. LECs grown on this surface were then stimulated with sHA on the luminal side by the addition of sHA dissolved in the growth medium at a final concentration ranging from 0 to 20 µg/mL. The impact of the sHA on the LECs was determined 48 h after seeding by measuring metabolic activity with the AlamarBlue assay which reacts to the reducing environment of viable cells and normalizing it to the number of cells on each surface as determined by the CyQuant assay. No effect in a concentration range of 0 to 20 µg/mL sHA was observed (Figure 2A).

Additionally, we compared sHA produced by enzymatic digestion with sHA created by heat treatment (Figures 2A,B). The relative metabolic activity of the LECs was not influenced significantly by the presence of either of the two sHA species in solution (Figures 2A,B). This was also true in control experiments carried out on cell culture plastic (see Supplementary Material) or in experiments employing thiolated sHA in solution (Figures 2C,D).

**Figure 3:**
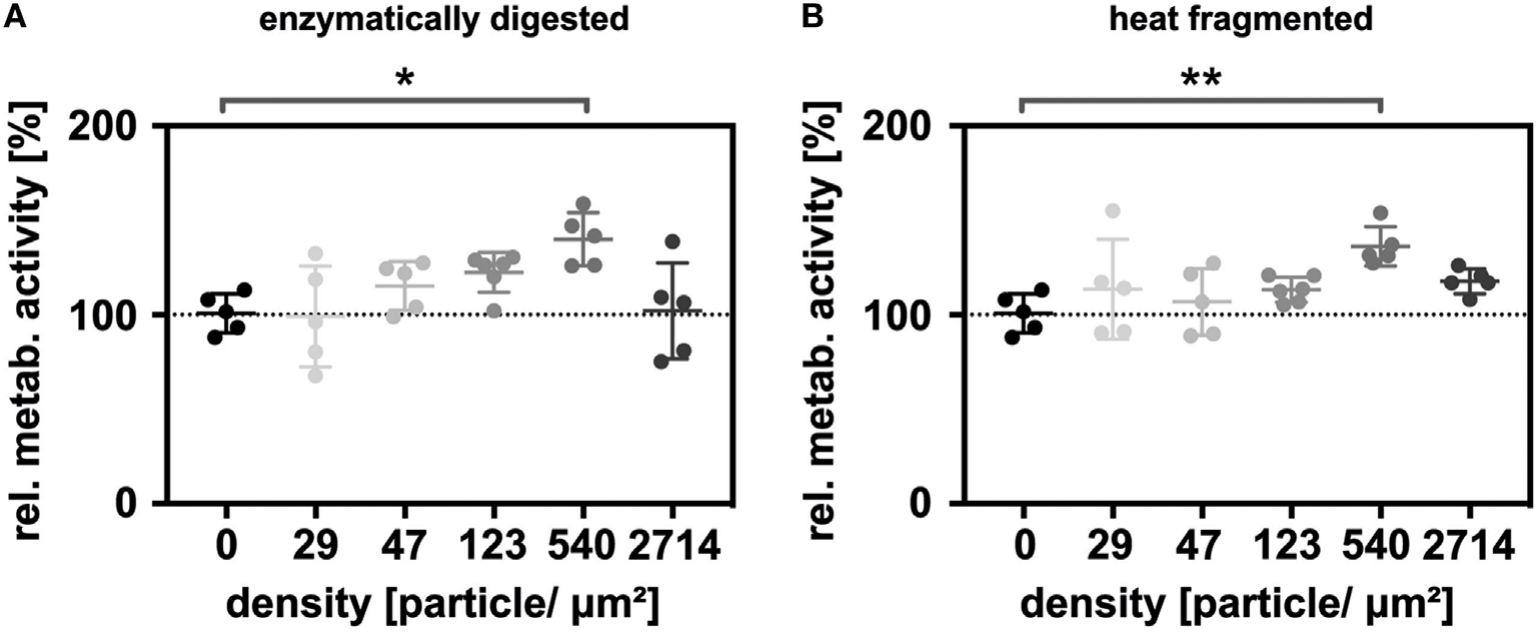
Immobilized HA oligosaccharides (sHA) exerting a biphasic effect on the metabolism of lymphatic endothelial cells (LECs). Graphs show the relative metabolic activity of LECs normalized to the relative amount of DNA on the surface for **(A)** the immobilized, enzymatically digested sHA and **(B)** the immobilized, heat-fragmented sHA species. To analyze the significance of the results, a Kruskal–Wallis test followed by the Dunn’s multiple comparison was carried out.

Next, the thiolated enzymatically digested and heat-fragmented sHA species were immobilized onto gold nanostructured glass slides at different particle densities. Five different nanoparticle densities (29, 47, 123, 540, 2,714 nanoparticles/μm^2^) were used in the experiments. Additionally, the surfaces were functionalized with click-PEG bearing c(RGDfE). The relative metabolic activity was again determined 48 h after seeding. Under these conditions, a biphasic effect on LEC metabolism was observed. For both sHA species, metabolism increased with increasing nanoparticle density, with maximal stimulation being observed at 540 particles/μm^2^, then decreased in response to even higher densities (Figure 3). The relative metabolic activity at a nanoparticle density of 540 nanoparticles/μm^2^ was significantly increased for both sHA species (Kruskal–Wallis test followed by the Dunn’s multiple comparison, *p* < 0.05). The level of significance was higher in the case of the sHA obtained through heat fragmentation compared with the enzymatically digested HA.

## Discussion

Here, we describe the development of adhesive surfaces that mimic specific aspects of the ECM of lymphatic capillaries, and enable the stimulation of LECs on either their luminal or basal side. Cyclic RGD was employed to facilitate cell attachment, which proved to be comparable to the adhesive surface provided by cell culture plastic. Due to the precise control of key parameters in our system, these surfaces provide a platform for systematically studying cellular responses to particular matrix conformations.

In this study, we have focused on using the multicomponent adhesive surfaces to analyze the relative metabolic activity of LEC cells in response to exposure to sHA. Normalization proved to be especially important when using the nanostructured surfaces, as different numbers of cells initially attached to the interfaces due to increased steric hindrance for high HA densities. Normalizing the metabolic activity to the amount of DNA on the corresponding surface ensured the comparability of the determined metabolic activity for the different surfaces.

No significant difference in the relative metabolic activity of LECs exposed in solution to any of sHA species used in these studies was observed. Thus, effects due to the sHA modification as well as the cleavage mechanism on the metabolic activity of LECs can be excluded. We have previously reported a biphasic effect on the proliferation of primary human LEC upon incubation with sHA (4, 8, and 13 disaccharide units) in an LYVE-1-dependent manner (Bauer et al., 2018). In these experiments, we observed a maximal proliferation-promoting effect for 5.0 µg/mL of sHA in solution. These data indicate that the metabolic activity of LECs is not coupled to their proliferation status. Further experiments will investigate which cellular HA receptors mediate the effects of tethered sHA on metabolism.

For all of our experiments, the influence of both the heat-fragmented sHA with an average weight of 20 kDa (roughly 50-mer) and the enzymatically digested sHA with an average weight of <10 kDa (about 25-mer) led to the same results. As sHA in solution had no impact on LEC metabolism, we conclude that the increased relative metabolic activity for immobilized sHA (540 nanoparticles/μm^2^) represents a specific response of the cells to the immobilized sHA. A conceivable explanation can be found in clustering of the HA receptors, which is postulated to be necessary to create a strong interaction between HA and its receptors (Lawrance et al., 2016). The interparticle distance of 540 nanoparticles/μm^2^ might create an HA density, which stimulates the clustering and thereby the metabolic activity. Our previously published data also show the proliferative response of LECs to sHA takes place within a narrow concentration range, which can be reflected in the specific response to immobilized HA (Bauer et al., 2018). Furthermore, the sHA used to stimulate the basal surface of the LECs was functionalized on its reducing end to immobilize it. As we saw, a specific response to this functionalized sHA, the stimulation of metabolism is not due to any interaction with the reducing end of the sHA molecule or the cleavage method used to prepare the sHA.

In summary, the specific increase in relative metabolic activity of primary human LEC cells in response to immobilized sHA species opens up new avenues to trigger and control lymphangiogenesis, for example, to stimulate wound healing after surgery.

## Author Contributions

HB and JS conceived and coordinated the study. CA, HB, and JS wrote the paper. CA and YM performed and analyzed the experiments. JB performed the MTT assay and the peptide screening. All authors reviewed the results and approved the final version of the manuscript.

## Conflict of Interest Statement

The authors declare that the research was conducted in the absence of any commercial or financial relationships that could be construed as a potential conflict of interest.
