# Supplementary material for: Effect of Co-presentation of Adhesive Ligands and Short Hyaluronan on Lymphendothelial Cells
Source: Front Bioeng Biotechnol. 2018 Mar 23;6:25. doi: 10.3389/fbioe.2018.00025 (PMC5876295; doi:10.3389/fbioe.2018.00025)
Supplement: Supplementary file 1 [file data_sheet_1.docx]

**SI – Effect of Co-presentation of Adhesive Ligands and Short Hyaluronan on Lymphendothelial Cells**

*Christiane H. Antoni, Yvonne McDuffie, Jochen Bauer, Jonathan P. Sleeman and Heike Boehm*

## Characterization of Modified sHA

To verify the chemical modification of the sHA an indirect method was used. Due to the large difference in mass between the used sHA species (10000 or 20000 g/mol) and the introduced cysteamine hydrochloride (113.61 g/mol) and the very similar chemical shift in NMR experiments the classical chemical analytic techniques – like NMR, MS and IR - cannot be used for the characterization. We showed a method to overcome this disadvantage (Minsky et al., 2016). Therefore we used quartz-crystal microbalance with dissipation monitoring (QCM-D) to observe the adsorption of the thiolated HA on a gold coated quartz crystal (QSX301, Q-Sense AB). The technique is based on the inverse piezoelectric effect. The oscillation frequency of a quartz crystal changes due to the adsorption of mass on its surface (Dixon, 2008; O'Sullivan et al., 1999; Sauerbrey, 1959). The experiments were performed with the QCM-D E4 (Q-Sense AB) and a custom made open-module, which enables the direct pipetting on top of the crystal. The crystal was equilibrated in 200 μL PBS before 200 μL of either a 0.4 g/L solution of end-thiolated sHA, unmodified sHA or a 100 μM solution of cysteamine hydrochloride in PBS was added to the crystal (fig. S1). When the equilibrium was reached, the solution was removed three times and the crystal was again equilibrated in PBS. The remaining change in frequency is related to absorbed molecules on the surface.


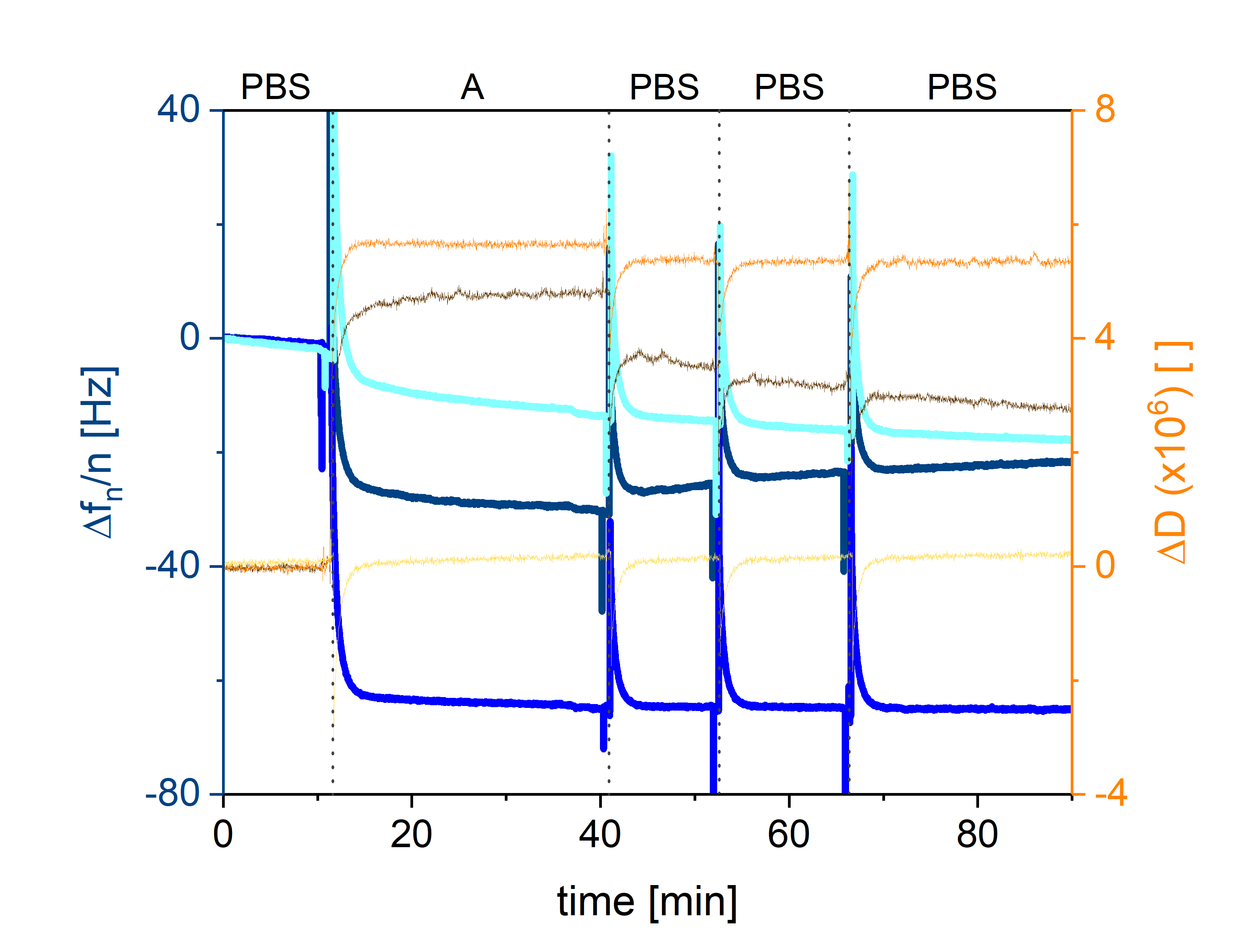


*Fig. S1: The chemical modification of sHA was verified using QCM-D. Compared are the adsorption of end-thiolated sHA* *(0.4 g/L, Δf7: blue, D: orange), unfunctionalized sHA (0.4 g/L, Δf7:dark blue, D: dark brown) and cysteamine hydrochloride (100 μm, Δf7: light blue, D: light brown) for the 7^th^ overtone on a gold surface: the system was equilibrated in PBS, then the three compounds were added in PBS. Afterwards, the crystal was washed three times with PBS (Antoni, 2017).*

Because of the correlation between change in frequency (Δf) and adsorbed mass, a larger change is expected for the modified sHA (found: Δ (Δf_7_) = -65 Hz; (Antoni, 2017)) compared to the cysteamine (found: Δ (Δf_7_) = ‑17 Hz; (Antoni, 2017)). Because the adsorption comes from the very strong interaction between thiol and gold, the unmodified HA cannot bind to the gold surfaces, and so no or only a small change in frequency should be observed (found: Δ (Δf_7_) = -22 Hz; (Antoni, 2017)). The significant larger change in frequency for the thiolated sHA verifies the successful chemical modification. For the preparation of the bifunctionalized surface the thiolated sHA is as well adsorpted to a gold surface in this case the gold nanoparticles. If the thiolated sHA was binding in the QCM-D experiment, it will also bind to the gold nanoparticles. We showed further characterization of the sHA layer on the gold surface to prove the adsorption by analysing the passivation properties of the different surfaces as well as its bioactivity (Antoni, 2017; Minsky et al., 2016).

## Selection of the Peptide for Surface Functionalization

To choose the optimal peptide to create an adhesive background on the surface, nanostructured surfaces with an interparticle distance of 20 nm were prepared and passivated with PEG2000-silane as described in the experimental section. The nanoparticles were functionalized with different peptides via thiol groups. To this end the peptides were diluted to a concentration of 50 μM and incubated with the surfaces (d = 22 mm, Menzel Glaeser) upside down on parafilm in an humidity chamber for 1.5 hrs. Afterwards, the surfaces were washed with ddH_2_O and dried under a stream of nitrogen.


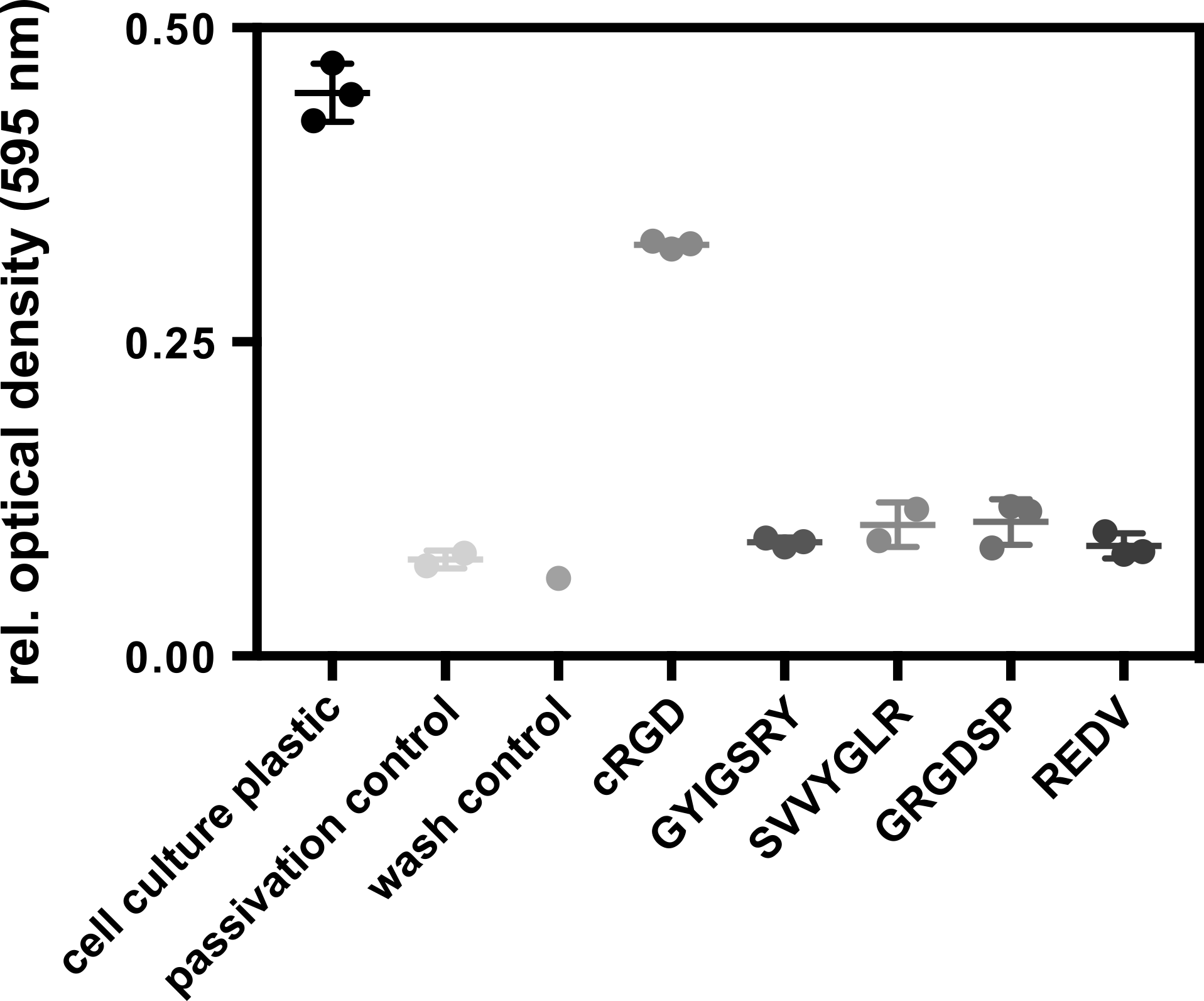


*Fig. S2: Nanostructured surfaces with an interparticle spacing of 20 nm with the indicated thiolated peptides were used to determine conditions required to achieve cell adhesion comparable to cell culture plastic. The MTT assay was used to determine LEC adhesion to the different surfaces.*

Adhesion of the cells to the surfaces was evaluated using the MTT assay. LECs were seeded at a density of 8000 cells/cm^2^. After 72 h incubation at 37°C, (3-(4,5-Dimethylthiazol-2-yl)2,5)diphenyltetrazolium bromide (Sigma-Aldrich) was added to a final concentration of 1 mg/mL. After incubation at 37°C for another 4 hours, medium was removed and 200 μl 0.04 M HCl in isopropanol was added. For quantification the relative optical density was measured at 595 nm (Fig. S2).

Adhesion of LECs to functionalized surfaces that was comparable to adhesion to cell culture plastic was only observed with the peptide cRGD. This peptide was therefore used for all further experiments.

## Optimisation of the Ratio of PEG_2000_-silane and alkyne-PEG_3000_-silane

In further experiments the optimal density of the peptide ligand was determined. Glass surfaces (d = 22 mm, Menzel Glaeser) were modified with click-PEG and functionalized with c(RGDfE)-KN_3_ as described in the experimental section. A ratio of alkyne-PEG_3000_-silane and PEG_2000_-silane of 1:10 and 1:100 was tested and compared. In wash control experiments the click reaction was carried without the alkyne species. LECs adhered equivalently to cell culture plastic and both ratios of alkyne-PEG_3000_-silane and PEG_2000_-silane (Fig. S2). Cells bound poorly to the corresponding wash controls, verifying effective passivation.


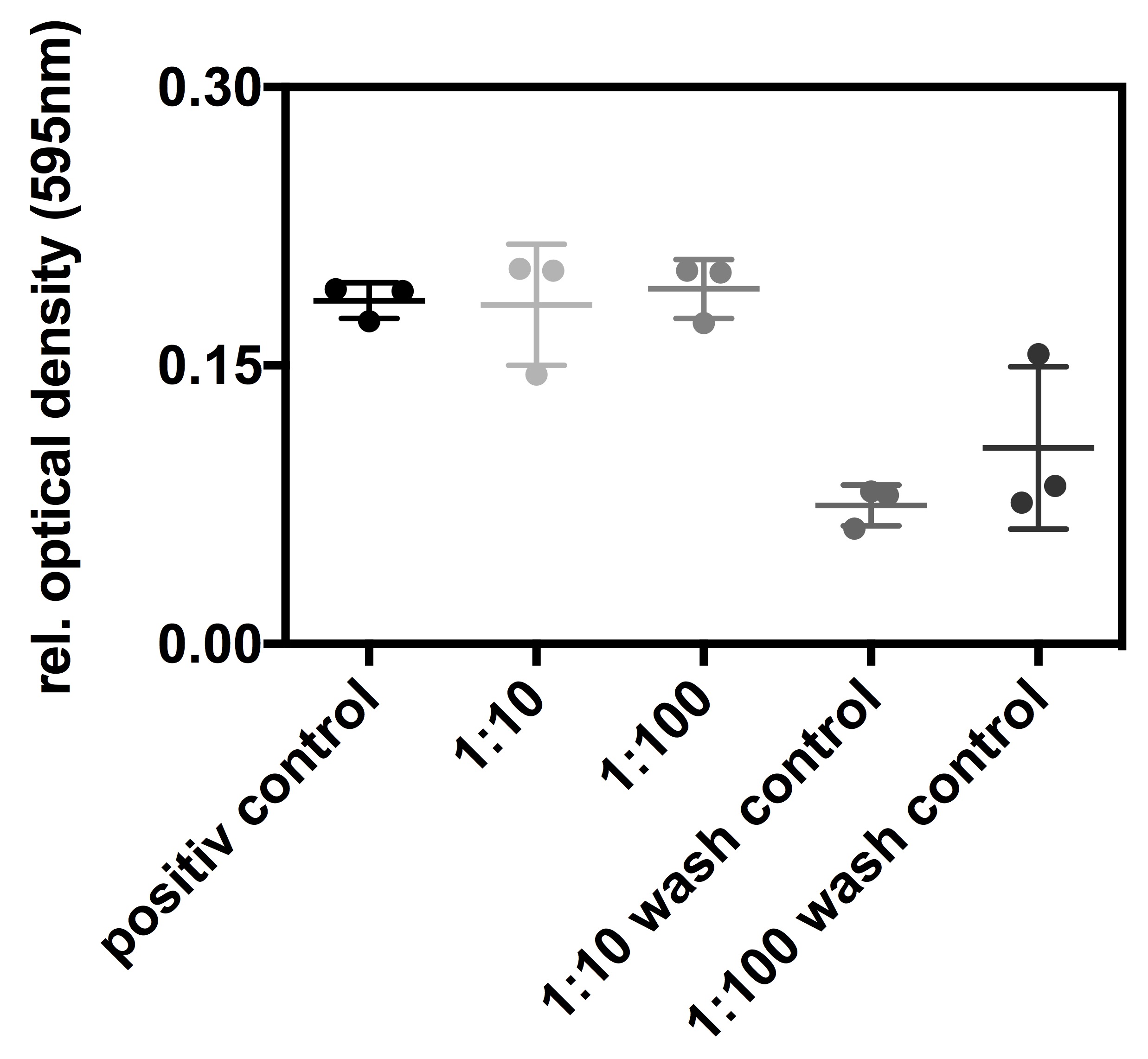


*Fig. S3: LEC adhesion to surfaces prepared with ratios of 1:10 and 1:100 of alkyne-PEG_3000_-silane and PEG_2000_-silane was measured by the MTT assay (relative optical density). Surfaces functionalized with c(RGDfE) was equivalent to adhesion to cell culture plastic (positiv control). In case of the wash controsl (pure PEG_2000_-silane) almost no cells adhered.*

Adhesion of the cells to the surfaces was evaluated using the MTT assay. LECs were seeded at a density of 8000 cells/cm^2^. After 72 h incubation at 37°C, (3-(4,5-Dimethylthiazol-2-yl)2,5)diphenyltetrazolium bromide (Sigma-Aldrich) was added to a final concentration of 1 mg/ml. After incubation at 37°C for another 4 hours, medium was removed and 200 μl 0.04 M HCl in isopropanol was added. For quantification the relative optical density was measured at 595 nm.

For all further experiments the 1:100 ratio was chosen to ensure that the surfaces retain sufficient space for orthogonal functionalization with nanoparticles and hyaluronan.

## Comparison of the Cell Morphology on the Used Surfaces

Because different surface modifications were used we compared the morphology of the cells on the different surfaces. Therefore cells were seeded on glass surfaces modified with click-PEG with and without the adhesive ligand cRGD and the bifunctionalized surface (Antoni, 2017). Compared were the wash control surface (PEG-passivated surface incubated with thiolated sHA and cRGD in absence of the reagents for the click reaction), the passivation control surface and cRGD functionalized surfaces (in the absence and presence of immobilised sHA). Therefore, phase contrast images were taken 48 hrs after seeding. The cells were fixed using a 4% solution of PFA (Alpha Aeser), afterwards washed with PBS and the cell membranes permeabilized for 3 min with 0.1% Triton X-100 (Sigma-Aldrich) in PBS. After 60 min incubation with 5% goat serum (Invitrogen) in PBS, the cells were incubated with Phalloidin/TRITC (1:100, Sigma-Aldrich) and DAPI (1:100, Sigma-Aldrich) on parafilm for 60 min. Then, the surfaces were mounted with Mowiol (Carl Roth) and pictures of the cells were taken using an upright, fluorescence microscope (Leica DM 6000B, 10x HCX PL APO). The phase contrast images were taken with an inverted microscope (Zeiss Observer Z1, 20x EC-Plan Neofluar, PH2).

*
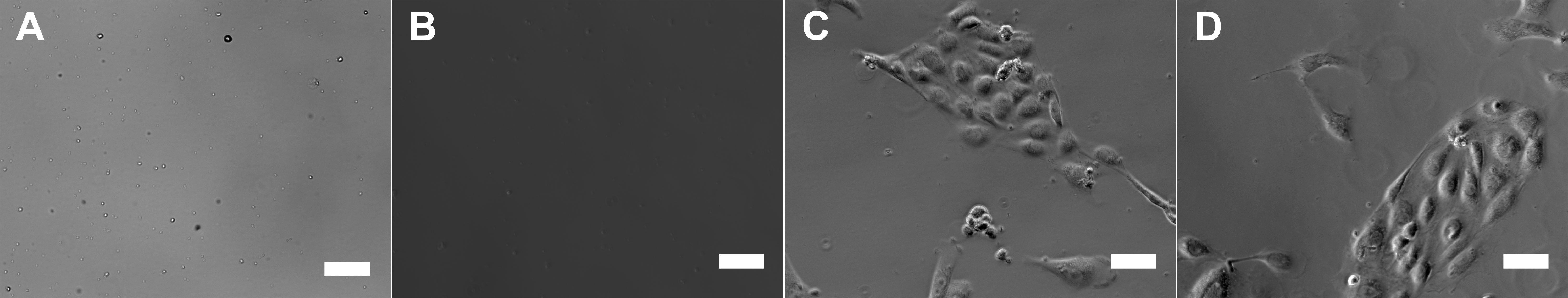
*

*Fig. S4: The comparison of the cell morphology on the different surfaces showed no visible difference. The cells were seeded on A the wash control, B the passivation control (no cell attachment), surfaces functionalized with click-PEG with cRGD (C) and bifunctionalized with cRGD and sHA (D) on a nanostructured surface with 540 nanoparticles/ μm^2^ (scale bar:50 μm).*

Well spreaded cells were found in the phase contrast images (fig. S4) as well as in the fluorescence images (fig. S5). In both cases no cell adhesion is found in the absence of cRGD. The wash control (fig. S4/5 A) showed that the cRGD cannot stick to the surfaces without performing the click reaction. Thus cell adhesion proves a successfully performed click reaction. In the passivation control, which is not incubated with either sHA nor cRGD, the performance of the PEG passivation itself is demonstrated (fig. S4/5 B). The comparison of the different adhesive surfaces showed no visible difference in morphology of the cells.


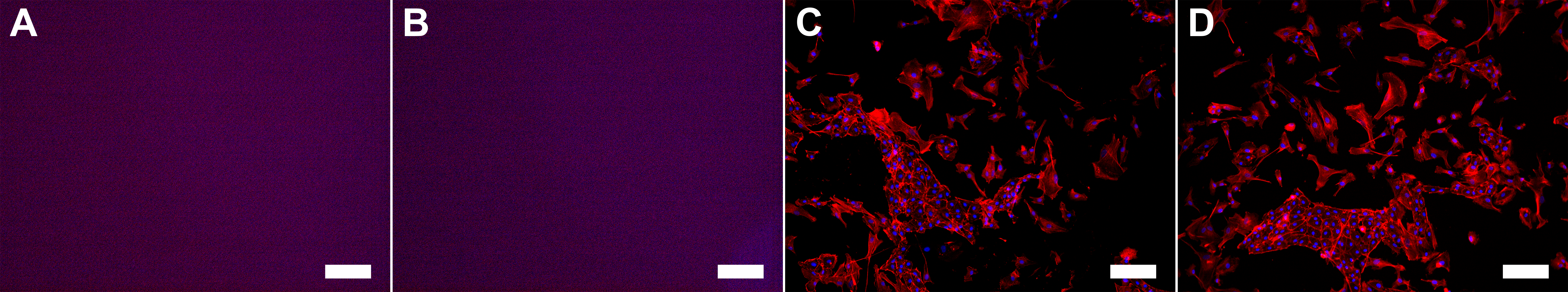


*Fig. S5: The comparison of the fluorescently labelled cells on the different surfaces showed only cell adhesion on surfaces in the presence of cRGD. The cells were seeded on different click-PEG surfaces: A incubated with thiolated sHA and cRGD but without nanoparticles and without click reaction (wash control); B click-PEG without cRGD (passivation control); C with cRGD without HA and D bifunctionalized with cRGD and sHA on a nanostructured surface with 540 nanoparticles/ μm^2^. The figure shows the overlay of the labelled actin filaments (red) and nuclei (blue; scale bar:150 μm).*

## Determination of the Relative Metabolic Activity on Cell Culture Plastic

LECs (4210 cells per well) were cultivated 96 well plates (GreinerBioOne). Each well contained 200 μL medium containing 0.0, 1.0, 2.5, 5.0 and 20.0 μg/mL of the HA species indicated. The cells were incubated for 46 hrs at 37°C and 5% CO_2_ then AlamarBlue and CyQuant assay were performed as described in Materials and Methods. The results showed that increasing concentration of enzymatically digested or heat fragmented sHA had no impact on the relative metabolic activity of the LECs (Fig. S6).

*
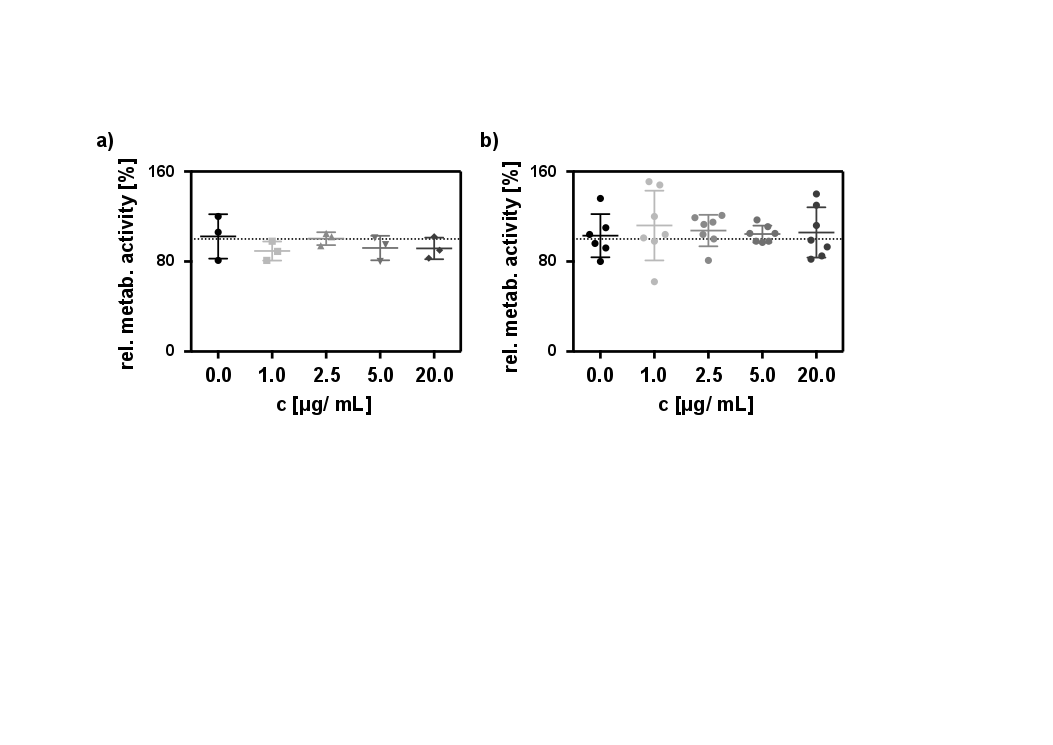
*

*Fig. S6: sHA in solution does not affect the relative metabolic activity of LECs on cell culture plastic. Data show the metabolic activity of LECs normalised to the amount of DNA a) using enzymatically digested sHA and b) a heat fragmented sHA. No significant difference between the control and the different concentrations of the HA species was found using the Kruskal-Wallis test followed by the Dunn's multiple comparison.*

## Control Experiments

Glass surfaces without nanoparticles were passivated with click PEG according the protocol described in the experimental section without CuAAC-mediated modification with c(RGDfE) (Fig. S6, A). To ensure that the c(RGDfE) did not stick to the PEG species by itself, CuAAC was performed on click-PEG passivated glass surfaces without copper, so that the azide and alkyne could not react (cRGD wash control, Fig. S5, B). Two positive controls were also performed in which cells were seeded on cell culture plastic (Fig. S5, C) or on the cRGD modified click-PEG glass surface without HA (Fig. S5, D).

**

*Fig. S7: The relative metabolic activity of LECs cultivated on either passivation control surfaces (A), c(RGDfE) wash control surfaces (B), cell culture plastic (C) or cRGD-modified click-PEG surfaces (D). The experiments were performed with a) HA in solution and b) immobilized on the surface.*

For A and B no cell adhesion was observed with the CyQuant assay, and so no metabolic activity could be determined. The comparison between C and D showed no significant difference between cell culture plastic and the click-PEG modified surface. Thus, no influence due to the surface itself on the cells was observed.

## REFERENCES

Antoni, Christiane (2017). *Chemically Modified Substrates to Probe Cell Behaviour in Wound Healing.* PhD Thesis. Heidelberg: Ruprecht-Karls Universität.

Dixon, M. C. (2008). Quartz Crystal Microbalance with Dissipation Monitoring: Enabling Real-Time Characterization of Biological Materials and Their Interactions. *Journal of Biomolecular Techniques* 19, 151–158.

Minsky, B. B., Antoni, C. H., and Boehm, H. (2016). Controlled Immobilization Strategies to Probe Short Hyaluronan-Protein Interactions. *Scientific reports* 6, 21608. doi: 10.1038/srep21608.

O'Sullivan, C. K., and Guilbault, G.G. (1999). Commercial quartz crystal microbalances - theory and applications. *Biosensors and Bioelectronics*, 663–670.

Sauerbrey, G. (1959). Verwendung von Schwingquarzen zur Wäigung dünner Schichten und zur Mikrowäigung. *Zeitschrift für Physik*, 206–222.
